# Polymorphisms of TNF-alpha (− 308), IL-1beta (+ 3954) and IL1-Ra (VNTR) are associated to severe stage of endometriosis in Mexican women: a case control study

**DOI:** 10.1186/s12905-022-01941-5

**Published:** 2022-08-26

**Authors:** Jennifer Mier-Cabrera, Oliver Cruz-Orozco, Julio de la Jara-Díaz, Oscar Galicia-Castillo, Mario Buenrostro-Jáuregui, Alicia Parra-Carriedo, César Hernández-Guerrero

**Affiliations:** 1 Division of Social Studies, Department of Health, Universidad Iberoamericana, Prolongación Paseo de la Reforma 880, Col. Lomas de Santa Fe, C.P. 01219 Álvaro Obregón, Mexico City, Mexico; 2 Medical Division, Department of Gynecology, Instituto Nacional de Perinatología “Isidro Espinosa de los Reyes”, C.P. 11000 Miguel Hidalgo, Mexico City, Mexico; 3 Division of Social Studies, Department of Psychology, Universidad Iberoamericana, C.P. 01219 Álvaro Obregón, Mexico City, Mexico

**Keywords:** Inflammation, Pro-inflammatory cytokines, Single nucleotide polymorphism, IL1RN*2, TNF*2, IL1B*2, Endometriosis stage IV

## Abstract

**Background:**

Endometriosis is an estrogen-dependent and chronic inflammatory disease affecting up to 10% of women. It is the result of a combined interaction of genetic, epigenetic, environmental, lifestyle, reproductive and local inflammatory factors. In this study, we investigated whether single nucleotide polymorphisms (SNPs) mapping to TNF-alpha (TNF, rs1800629) and IL-1beta (IL1B, rs1143634) and variable number tandem repeat polymorphism mapping to IL1-Ra (IL1RN intron 2, rs2234663) genetic loci are associated with risk for endometriosis in a Mexican mestizo population.

**Methods:**

This study included 183 women with confirmed endometriosis (ENDO) diagnosed after surgical laparoscopy and 186 women with satisfied parity and without endometriosis as controls (CTR). PCR/RFLP technique was used for genotyping SNPs (rs1800629 and rs1143634); PCR for genotyping rs2234663.

**Results:**

We found no statistical differences in age between groups nor among stages of endometriosis and the CTR group. We observed no difference in genotype and allele frequencies, nor carriage rate between groups in none of the three studied polymorphisms. The prevalence of TNF*2-allele heterozygotes (p = 0.025; OR 3.8), TNF*2-allele (p = 0.029; OR 3.4), IL1B*2-allele heterozygotes (p = 0.044; OR 2.69) and its carriage rate (p = 0.041; OR 2.64) in endometriosis stage IV was higher than the CTR group. Surprisingly, the carriage rate of IL1RN*2-allele (ENDO: p = 0.0004; OR 0.4; stage I: p = 0.002, OR 0.38; stage II: p = 0.002, OR 0.35; stage III: p = 0.003, OR 0.33), as well as the IL1RN*2-allele frequencies (ENDO: p = 0.0008, OR 0.55; I: p = 0.037, OR 0.60; II: p = 0.002, OR 0.41; III: p = 0.003, OR 0.38) were lower than the CTR group. Women with endometriosis stage IV (severe) had frequencies more alike to the CTR group in the IL1RN*2 allele frequency (31.2% vs. 27.2%) and carriage rate (37.5% vs. 41.9%).

**Conclusion:**

Although these polymorphisms are not associated with the risk of endometriosis, Mexican mestizo women with severe stage of endometriosis have higher frequencies of TNF*2-, IL1B*2- and IL1RN*2-alleles, which may explain a possible correlation with disease severity rather than predisposition or risk.

## Background

Endometriosis is recognized as an estrogen-dependent and chronic inflammatory gynecological disease characterized by the presence and growth of endometrial-like tissue outside the uterine cavity. It affects at least 10% of women of reproductive age, leading to infertility and symptoms such as chronic pelvic pain, dysmenorrhea, dyspareunia, dysuria, dyschezia and fatigue [1]. Although several hypothesis and multiple factors have been proposed trying to explain its origin, the etiopathogenesis of endometriosis is still unknown. Being such a complex gynecological disease [2] different new theories have been proposed trying to explain its physiopathology nature. The “bacterial contamination theory” [3] suggests that recognition of bacteria could elicit the immune response (inflammation and dysregulation), playing a key role in its pathogenesis as endometriosis appears to be associated with elevated levels of different pathogenic species. The genetic-epigenetic (G/E) theory [4] proposes that after the accumulation of several cellular G/E events, the cell crosses a limit threshold which gives arise to a number of alterations. Epigenetics changes have been reported in ectopic endometrium related to inflammation, estrogen and progesterone receptors.

In this respect, non-immune and immune cells in the peritoneal microenvironment have been identified as the main source of estrogens, prostaglandins, and pro-inflammatory cytokines [5–9]. Hence, local inflammatory cytokine and prostaglandin production, immune cell infiltration, estrogen dominance, progesterone resistance, chronic local inflammation and oxidative stress are correlated and contribute to the central processes leading to pain, remodeling of neighboring tissues, fibrosis, adhesion formation, and infertility. Also, an aberrant immune system seems to play a key role, as it appears that women with endometriosis are more susceptible to autoimmune disorders (systemic lupus erythematous, Sjögren syndrome, rheumatoid arthritis, celiac disease, multiple sclerosis and inflammatory bowel disease) [10, 11] compared to general female population.

Inflammation is influenced by genetic susceptibility. Inflammatory cytokine genes polymorphisms have been subject of study trying to explain the etiology of gynecological (leiomyomas) [12, 13] and non-gynecological pathologies [14–16]. Although the contribution of genetics is well supported by many studies, they have not provided a simple and unambiguous answer to the etiology of endometriosis [17, 18].

Different gene loci have been identified as risk factors in endometriosis, including those related to growth factors, matrix remodeling, hormone receptors and metabolism, adhesion molecules, oxidative stress, cytokines, and inflammation [19]. The frequency distribution of gene polymorphisms varies according to the ethnic component of each human subpopulation, which partly explains the predisposition to disease and/or response to nutrients/pharmacological treatment [20, 21]. Tumor necrosis factor-alpha (TNF-α) and interleukin-1 beta (IL-1β) are the first cytokines synthesized during the inflammatory process, while interleukin-1 Receptor antagonist (IL-1Ra) modulates and inhibits IL-1β activity [22].

The TNF-α gene (TNFA; 6p21.31) displays several single nucleotide polymorphisms (SNP) [23]. Specifically, the SNP in the promoter region (position G-308A) has been identified with an increased synthesis of TNF-α by carriers of the mutated allele (TNF*2) [24, 25]. The IL-1β gene (IL1B; 2q12.21) displays SNPs in the promoter region (T-31C, C-511 T) [26] and in the exon 5 (C+3954T) [27]. The latter has been identified with an increased production of this cytokine [28, 29], and it is associated with inflammatory diseases [30, 31]. The IL-1RA gene (IL1RN; 2q14.21) displays a variable number tandem repeat (VNTR) polymorphism in the intron 2 caused by 86 bp. There are six alleles according to the number of the 86-bp repeats: allele 1 (IL1RN*1) is the most common and has four repeats followed by allele 2 (IL1RN*2) with two repeats and three non-common alleles that have three (IL1RN*3), five (IL1RN*4) and six (IL1RN*5) repeats, respectively [32].

Data reported in the literature have associated endometriosis with several cytokine genes displaying both positive and negative associations [19, 33, 34]. Women with endometriosis might have a particular profile of cytokine polymorphisms, which might well determine them to respond with a greater inflammatory intensity, being this directly responsible of the biological alterations and symptoms suffered by this group of women. The aim of this study was to investigate the association of TNF-α (G-308 A), IL-1β (C+3954T) and IL1-Ra intron 2 VNTR polymorphisms with the risk of endometriosis in Mexican mestizo women.

## Methods

### Study design and patient population

In this case control study, we enrolled 369 adult women from a tertiary hospital in Mexico City. All women with infertility who attended the Department of Infertility and Sterility at the Instituto Nacional de Perinatología “Isidro Espinosa de los Reyes” (INPer) were considered eligible. The case group, called “endometriosis group” (ENDO), included 183 women diagnosed with endometriosis after undergoing laparoscopic surgery. Endometriosis staging was done according to the revised American Society for Reproductive Medicine (r-ASRM) staging score [35]. We did not include women that were diagnosed with pelvic inflammatory disease and those whose pain or infertility was due to other medical issues but endometriosis.

On the other hand, we invited all fertile women who attended the Department of Family Planning at INPer for bilateral tubal occlusion surgery as a definite contraceptive method to participate as "controls". The inclusion criteria were women without any apparent clinical symptom nor visual presence of endometriosis confirmed during the surgical procedure. The "control group" (CTR) included 186 women without endometriosis and confirmed fertility. We did not include fertile women that were diagnosed with pelvic inflammatory disease, endometriosis, or a medical history of myomas.

The Institutional Review Board and Ethics Committee of the INPer approved the study protocol (212250-06081). All procedures concerning this work comply with the Declaration of Helsinki. All women that accepted to participate were informed about the objectives and outcomes of the study and provided their written informed consent.

### Blood samples collection and DNA extraction

Peripheral venous blood samples were collected in 7-mL heparin tubes (Becton Dickinson Vacutainer Systems, Franklin Lakes, NJ, USA) and taken to the laboratory immediately for DNA extraction. Genomic DNA was isolated from whole blood (100 µL) using 1 ml of the DNAzol Reagent (Invitrogen, ThermoFisher Scientific, Waltham, MA, USA) according to the manufacturer’s instructions. The isolated DNA was stored at − 20 °C until it was used for the polymerase chain reaction (PCR) experiments.

### *TNF*, *IL1B* and *IL1RN* genotyping

TNF-α (G-308A; rs1800629) and IL-1β (C+3954T, rs1143634) SNPs were determined by the polymerase chain reaction-restriction fragment length polymorphism (PCR-RLFP) method using the restriction enzymes NcoI [36] and TaqI [37], respectively, as described elsewhere. IL-1Ra intron 2 (VNTR; rs2234663) alleles were determined using a PCR protocol [38], as described elsewhere. PCR reagents (10X PCR Buffer, MgCl_2_, Taq DNA polymerase, and dNTPs) were purchased from Invitrogen (ThermoFisher Scientific, Waltham, MA, USA).

Amplification of the genomic fragments where the polymorphic sites are located was carried out using the primers and PCR settings described in Table 1. For each PCR amplification protocol, we used 50 ng template DNA, 1.5 mM MgCl2, 1 Unit Taq DNA polymerase, 20 pmol of each primer, 0.2 mM of each dNTP and PCR grade water in a total reaction volume of 25 µl. All reactions were performed in a Mastercycler gradient thermal cycler (Eppendorf Scientific, Hamburg, Germany).

**Table 1:** Primers and PCR settings used for genotyping the cytokines polymorphisms

	TNF-α (rs1800629)	IL-1β (rs1143634)	IL-1Ra (rs2234663)
Primers	F:AGGCAATAGGTTTTGAGGGCCAT	F:CTCAGGTGTCCTCGAAGAAATCAAA	F:TCCTGGTCTGCAGGTAA
	R:TCCTCCCTGCTCCGATTCCG	R:GCTTTTTTGCTGTGAGTCCCG	R:CTCAGCAACACTCCTAT
Cycles	35	35	35
Initial denaturation	94 °C for 5 min	94 °C for 5 min	96 °C for 1 min
Denaturation	94 °C for 30 s	94 °C for 30 s	94 °C for 1 min
Annealing	60 °C for 30 s	55 °C for 30 s	62 °C for 1 min
Elongation	72 °C for 30 s	72 °C for 30 s	72 °C for 1 min
Final extension	72 °C for 5 min	72 °C for 5 min	72 °C for 7 min

### *TNF* (G-308A; rs1800629) RFLP

We digested 10 µL of PCR product (107 bp) with 4 units of NcoI restriction enzyme (Roche Molecular Biochem, Mannheim, Germany) during 24 h at 37 °C. Digestion product was analyzed by electrophoresis in a 4% agarose gel stained with ethidium bromide and visualized in an Epichemi^3^ Darkroom Transilluminator (UVP Inc., Upland, California, USA). The identification of two bands of 87 bp and 20 bp revealed TNF*1 allele; meanwhile, a single 107 bp band revealed TNF*2 allele.

### *IL1B* (C+3954T, rs1143634) RFLP

We digested 10 µL of PCR product (182 bp fragment) with 7 units of TaqI restriction enzyme (Roche Molecular Biochem, Mannheim, Germany) during 24 h at 65 °C. Digestion product was analyzed by electrophoresis in a 6% acrylamide gel stained with ethidium bromide and visualized using an Epichemi^3^ Darkroom Transilluminator (UVP Inc. Upland, CA, USA). The identification of two bands of 97 bp and 85 bp revealed the IL1B*1 allele; meanwhile, a single band of 182 bp revealed the point mutation corresponding to the IL1B*2 allele.

### *IL1RN* (rs2234663) VNTR

PCR product (20 µL) was analyzed by electrophoresis in a 2% agarose gel stained with ethidium bromide and visualized using an Epichemi^3^ Darkroom Transilluminator (UVP Inc. Upland, CA, USA). The identification of 412 bp, 240 bp, 326 bp, 498 bp, and 584 bp fragments corresponded to alleles 1, 2, 3, 4 and 5, respectively.

### Statistical analyses

All statistical analyses were assessed using the software SigmaStat v. 3.1 (Systat Software Inc., CA, USA). Age and obstetric characteristics were compared using Student's t, U-Mann Whitney, and ANOVA of Kruskall-Wallis tests, where applicable. Allele frequencies, genotype frequencies and carriage rate were computed. The polymorphisms were tested for Hardy–Weinberg equilibrium by the goodness-of-fit χ2 test. The χ2 test was used to examine the differences of allele and genotype frequencies, as well as carriage rate between groups. The risk associations for endometriosis were estimated by the odds ratio (OR) with 95% confidence interval (95% CI) and a p-value < 0.05 was considered statistically significant.

## Results

All women included in the CTR (n = 186) and ENDO (n = 183) group self-reported their ethnical origin as Mexican mestizo as their parents and grandparents were born in Mexico. All of them had middle educational (< 13 years) and socioeconomical status and lived in Mexico City or its surroundings. According to the rASRM classification, 63 (34.4%) women had endometriosis stage I (minimal), 54 (29.5%) stage II (mild), 117 (63.9%) stages I–II (minimal/mild), 42 (23.0%) stage III (moderate), 24 (13.1%) stage IV (severe) and 66 (36.1%) stages III–IV (moderate/severe). There were no statistically significant differences in the mean age between the CTR (33.8 ± 3.2 years) and ENDO (32.7 ± 2.5 years) group nor among rASRM stages of endometriosis and the control group (data not shown). Conversely, obstetric characteristics were significantly different between groups (p < 0.05). While the CTR group reported a median of three pregnancies, one vaginal delivery, one Cesarean delivery and zero abortions, the ENDO group reported zero pregnancies.

The genotype and allele frequency distribution of the TNF-α (− 308) polymorphism among the 369 women from the ENDO and CTR group is described in Table 2.

**Table 2:** Genotype and allele frequencies of the TNF-α − 308 polymorphism between women with and without endometriosis

TNF-α	n	Genotype n (%)			Allele n (%)	
		*1*1	*1*2	*2*2	1	2
Women without endometriosis	186	171 (91.9)	15 (8.1)	0 (0.0)	357 (95.7)	15 (4.3)
Women with endometriosis	183	159 (86.9)	24 (13.1)	0 (0.0)	342 (93.4)	24 (6.6)
Stage I	63	54 (85.7)	9 (14.3)	0 (0.0)	117 (92.9)	9 (7.1)
Stage II	54	48 (88.8)	6 (11.2)	0 (0.0)	102 (94.4)	6 (5.6)
Stage I/II	117	102 (87.2)	15 (12.8)	0 (0.0)	219 (93.6)	15 (6.4)
Stage III	42	39 (92.9)	3 (7.1)	0 (0.0)	81 (96.4)	3 (3.6)
Stage IV	24	18 (75.0)	6 (25.0)^1^	0 (0.0)	42 (87.5)	6 (12.5)^2^
Stage III/IV	66	57 (86.4)	9 (13.6)	0 (0.0)	123 (93.2)	9 (6.8)

^1^*x*^2^ = 5.02, p = 0.025, OR = 3.8 (95% CI 1.31–11.01) versus women without endometriosis

^2^*x*^2^ = 4.75, p = 0.029, OR = 3.4 (95% CI 1.25–9.23) versus women without endometriosis

Allele and genotype frequencies in the study population were in Hardy–Weinberg equilibrium (p > 0.05). The distribution of TNF*1-allele homozygotes (p > 0.05) and TNF*2-allele heterozygotes (TNF*1/TNF*2) (p > 0.05) genotype frequencies was not statistically significant between groups. More than 85% of women in both groups were TNF*1 homozygotes. However, according to the r-ASRM staging, the prevalence of TNF*2-allele heterozygotes in stage IV was the only statistically different (p = 0.025) when compared to the CTR group. We did not identify the TNF*2-allele homozygote in our sample. A similar pattern was observed when we analyzed the TNF*1 and TNF*2 allele frequencies. No statistically significant difference was observed between groups (p > 0.05); except when endometriosis stage IV (p = 0.029) was compared to the CTR group. We also analyze the genotype and allele frequency of this polymorphism by grouping together stages I/II and stages III/IV and comparing them to the CTR group. However, no differences were found. The associations analysis suggests an approximate fourfold increased risk for women with endometriosis stage IV with TNF*2-allele heterozygote genotype and an approximate 3.5-fold increased risk with TNF*2-allele.

The genotype and allele frequencies distribution and the carriage rate of the IL-1β (+ 3954) polymorphism among the 369 women from the ENDO and CTR group is showed in Table 3.

**Table 3:** Genotype and allele frequencies of the IL-1β (+ 3954) polymorphism between women with and without endometriosis

IL-1β	n	Genotype n (%)				Allele n (%)	
		*1*1	*1*2	*2*2	*1*2 + *2*2	1	2
Women without endometriosis	186	135 (72.6)	46 (24.7)	5 (2.7)	51(27.4)	316 (84.9)	56 (15.1)
Women with endometriosis	183	129 (70.5)	47 (25.7)	7 (3.8)	54 (29.5)	305 (83.3)	61 (16.7)
Stage I	63	51 (80.9)	9 (14.3)	3 (4.8)	12 (19.0)	111 (88.1)	15 (11.9)
Stage II	54	36 (66.7)	16 (29.6)	2 (3.7)	18 (33.3)	88 (81.5)	20 (18.5)
Stage I/II	117	87 (74.4)	25 (21.4)	5 (4.3)	30 (25.6)	199 (85.0)	35 (15.0)
Stage III	42	30 (71.4)	11 (26.2)	1 (2.4)	12 (28.6)	71 (84.5)	13 (15.5)
Stage IV	24	12 (50.0)	11 (45.8)^1^	1 (4.2)	12 (50.0)^2^	35 (72.9)	13 (27.1)
Stage III/IV	66	42 (63.6)	22 (33.3)	2 (3.0)	24 (36.4)	106 (80.3)	26 (19.7)

^1^*x*^2^ = 4.03, p = 0.044, OR = 2.69 (95% CI 1.11–6.51) versus women without endometriosis

^2^*x*^2^ = 4.14, p = 0.041, OR = 2.64 (95% CI 1.11–6.27) versus women without endometriosis

Allele and genotype frequencies in the study population were in Hardy–Weinberg equilibrium (p > 0.05). The distribution of the three genotype frequencies (IL1B*1-allele homozygote, IL1B*2-allele heterozygote and IL1B*2-allele homozygote) and the carriage rate was not statistically significant between groups (p > 0.05). We identified that > 70% of women in both groups were IL1B*1 homozygotes. On the other hand, women with endometriosis stage I showed the highest frequency (80%) of this genotype, while those with endometriosis stage IV, the lowest (50%). Although the IL1B*2-allele heterozygote genotype frequency was similar between groups, we only found a statistically significant difference between women with stage IV endometriosis and the CTR group (p = 0.044). The IL1B*2-allele homozygote was found in a very low frequency (< 5%) in both groups and in all four stages of endometriosis. When we analyzed the carriage rate of IL1B*2 allele (*1*2 + *2*2), we found a statistically significant difference between women with endometriosis stage IV and the CTR group (p = 0.041). A similar pattern was observed when we analyzed the IL1B*1 and IL1B*2 allele frequencies. Regarding the IL1B*2-allele, its frequency was very alike between groups (15.1% vs. 16.7%) and no statistically significant difference was found (p > 0.05). Likewise, we found no statistical difference among the four stages of endometriosis and the CTR group, nor stages I/II, III/IV and the CTR group. The associations analysis suggests an approximate threefold increased risk for women with endometriosis stage IV with IL1B*2-allele heterozygote genotype and a tendency to a twofold increased risk with IL1B*2-allele [p = 0.056; OR 2.09; 95%CI (1.04–4.02)].

The genotypes and alleles frequencies distribution and the carriage rate of IL-1Ra (86 bp, VNTR) polymorphism is described in Table 4.

**Table 4:** Genotype and allele frequencies of the IL-1 Ra VNTR polymorphism between women with and without endometriosis

IL-1Ra	n	Genotype n (%)							Allele frequency n (%)			
		*1*1	*1*2	*1*3	*1*4	*2*2	*1*3 + *1*4	*1*2 + *2*2	1	2	3	4
Women without endometriosis	186	90 (48.4)	55 (29.6)	10 (5.4)	8 (4.3)	23 (12.4)	18 (9.7)	78 (41.9)	253 (68.0)	101 (27.2)	10 (2.7)	8 (2.1)
Women with endometriosis	183	129 (70.5)	25 (13.7)	4 (2.2)	5 (2.7)	20 (10.9)	9 (4.9)	45 (24.6)^1^	292 (79.8)	65 (17.8)^7^	4 (1.1)	5 (1.3)
Stage I	63	45 (71.4)	6 (9.5)	2 (3.2)	1 (1.6)	9 (14.3)	3 (4.8)	15 (23.8)^2^	99 (78.6)	24 (19.0)^8^	2 (1.6)	1 (0.8)
Stage II	54	39 (72.2)	9 (16.7)	1 (1.8)	2 (3.7)	3 (5.6)	3 (5.6)	12 (22.2)^3^	90 (83.3)	15 (13.9)^9^	1 (0.9)	2 (1.9)
Stage I/II	117	84 (71.8)	15 (12.8)	3 (2.6)	3 (2.6)	12 (10.3)	6 (5.1)	27 (23.1)^4^	189 (80.8)	39 (16.7)^10^	3 (1.3)	3 (1.3)
Stage III	42	31 (73.8)	7 (16.7)	2 (4.8)	0 (0.0)	2 (4.7)	2 (4.8)	9 (21.4)^5^	71 (84.5)	11 (13.1)^11^	2 (1.2)	0 (0.0)
Stage IV	24	14 (58.3)	3 (12.5)	1 (4.2)	0 (0.0)	6 (25.0)	1 (4.2)	9 (37.5)	32 (66.7)	15 (31.2)	1 (2.1)	0 (0.0)
Stage III/IV	66	45 (68.2)	10 (15.2)	3 (4.5)	0 (0.0)	8 (12.1)	3 (4.5)	18 (27.3)^6^	103 (78.0)	26 (19.7)^12^	3 (2.3)	0 (0.0)

^1^*x*^2^ = 16.6, p = 0.0004, OR 0.40 (95% CI 0.25–0.63), versus women without endometriosis

^2^*x*^2^ = 9.3, p = 0.002, OR 0.38 (95% CI 0.19–0.74), versus women without endometriosis

^3^*x*^2^ = 9.4, p = 0.002, OR 0.35 (95% CI 0.17–0.72), versus women without endometriosis

^4^*x*^2^ = 14.8, p = 0.0001, OR 0.37 (95% CI 0.21–0.62), versus women without endometriosis

^5^*x*^2^ = 8.6, p = 0.003, OR 0.33 (95% CI 0.15–0.74), versus women without endometriosis

^6^*x*^2^ = 6.77, p = 0.009, OR 0.46 (95% CI 0.24–0.86), versus women without endometriosis

^7^*x*^2^ = 11.17, p = 0.0008, OR 0.55 (95% CI 0.39–0.79), versus women without endometriosis

^8^*x*^2^ = 4.32, p = 0.037, OR 0.60 (95% CI 0.36–1.00), versus women without endometriosis

^9^*x*^2^ = 9.47, p = 0.002, OR 0.41 (95% CI 0.23–0.75), versus women without endometriosis

^10^*x*^2^ = 10.5, p = 0.001, OR 0.51 (95% CI 0.34–0.76), versus women without endometriosis

^11^*x*^2^ = 8.78, p = 0.003, OR 0.38 (95% CI 0.19–0.76), versus women without endometriosis

^12^*x*^2^ = 3.86, p = 0.049, OR 0.63 (95% CI 0.388–1.03), versus women without endometriosis

Allele and genotype frequencies in the study population were not in Hardy–Weinberg equilibrium (p < 0.05). The IL1RN*1-allele homozygote genotype was the most common of all in the ENDO group as well as in stages I-III (> 70%), but not for stage IV (58%). Conversely, this genotype was only present in 48% of women without endometriosis. The frequency of the IL1RN*1/IL1RN*2 genotype was higher in the CTR group (29%). The IL1RN*1/IL1RN*3 and IL1RN*1/IL1RN*4 genotypes showed very low frequencies (< 6%) and carriage rate (*1*3 + *1*4; < 10%) in both groups and in all four stages of endometriosis. However, we did not find IL1RN*1/IL1RN*4 genotype in women with endometriosis stage III and IV. The IL1RN*2-allele homozygote genotype frequency was very alike in both groups and no statistical difference was found among groups when compared to the CTR group. When we analyze the carriage rate of IL1RN*2-allele (*1*2 + *2*2), the ENDO group and the four stages of endometriosis had lower frequencies than the CTR group. All groups showed statistical difference, except for endometriosis stage IV. Surprisingly, all OR values were below 0.63 (a protective effect), except again for stage IV (OR 0.83). We found all alleles in both groups, except for IL1RN*5. The most common alleles were IL1RN*1 (68% and 80% in women without and with endometriosis, respectively) and IL1RN*2 (27% and 18% in women without and with endometriosis, respectively), as reported in the literature. Surprisingly, we observed a higher frequency of IL1RN*1 in women with endometriosis and a higher frequency of IL1RN*2 in the CTR group, which was statistically different (p < 0.0008). The frequency of IL1RN*3 and IL1RN*4 was < 3% in both groups. The ENDO group and endometriosis stages I-III had similar frequencies of IL1RN*1-allele (79.8–84.5%); however, this phenomenon was not observed in endometriosis stage IV, which frequency was very alike to that of the CTR group (67 vs. 68%). When we analyzed the IL1RN*2-allele frequency according to rASRM staging, all endometriosis stages showed statistical difference when compared to the CTR group, except for endometriosis stage IV (p > 0.05). Finally, we also found statistical differences in both carriage rate and IL1RN*2-allele frequency in both stage I/II and stage III/IV when compared to the CTR group.

## Discussion

Endometriosis is a multifactorial disease where inflammation is actively involved in the initiation, establishment, and development of ectopic endometrial tissue in the peritoneal cavity. This process is highlighted by the involvement of pro-inflammatory cytokines synthesized by immune and non-immune cells present in the peritoneal microenvironment. In this study, we investigated the association of TNF-α (G-308A), IL-1β (C+3954T) and IL-1Ra (intron 2, VNTR) polymorphisms and endometriosis in Mexican mestizo women from Mexico City and its surroundings.

Positive (− 1031), negative (− 308, − 238) or ambiguous (− 863, − 857) associations [39] have been reported between TNF-α polymorphisms and endometriosis. Specifically, the − 308 polymorphism has been evaluated in nine studies [Asia (6), Europe (1) and Australia (1)], including ours (Mexico), and two systematic reviews and meta-analysis in Asians [40, 41] yielding negative associations in all cases. Regionally and geographically, the frequencies of TNF*2-allele have been shown to significantly differ. Wiezer (Austria) [42] and Hsieh (China) [43] described very similar genotype and allele frequencies in contrast to Zhao (Australia) [44], who studied haplotypes and found no association. Our results were very similar to that found by Lee (Korea) [45] and Babaabasi (Iran) [46]. Although the latter identified the TNF*2-allele homozygote (< 7%), we did not, as it appears to be very low or even absent in the American mestizo or Amerindian population [47, 48]. The higher prevalence of the TNF*2 heterozygote genotype observed in endometriosis stage IV (12.5%) might be related to an increased fashion synthesis of TNF-α by immune and non-immune cells, promoting the development and maintenance of endometriosis through the expression of different molecules related to growth, adhesion, maintenance, and survival.

There is still a scientific debate regarding the association of different IL-1β SNPs and endometriosis. All five studies (Turkey, Taiwan, China, Austria, Mexico) that have investigated the relationship between the C+3954T SNP and endometriosis found a negative association. The allele frequencies identified range from 1 to 43% in women with endometriosis [49–52]. Attar [51], like us, identified an increased frequency in endometriosis stage IV (27%, p = 0.056), which could be related to an increased synthesis of IL-1β by peritoneal immune and non-immune cells that can induce the expression of different molecules involved in the immunological dysfunctions contributing to the establishment and progression of the disease.

The regulation of IL-1β by IL-1Ra should be coordinated during inflammation to cease the immune response and limit the potential for immunopathology [53, 54]. Four studies (Taiwan, Korea, China and Mexico) [49, 52, 55], have evaluated the association between IL1-Ra VNTR polymorphism and endometriosis. Unlike them, who found IL1RN*1-allele homozygote in more than 84% and 92% of women with and without endometriosis, we observed it in 70% and 48%, respectively. Like Hsieh [49] and Wen [52], we found the IL1RN*1/IL1RN*2-allele heterozygote in approximately 10% of women with endometriosis. Nevertheless, this genotype was present in 30% of Mexican women without endometriosis, while they found it in < 6%. Surprisingly, we found the highest prevalence of the IL1RN*2-allele homozygote genotype (10% vs. 1%) and the IL1RN*2-allele in Mexican women with (18% vs. < 7%) and without endometriosis (27% vs. < 4%). Although Wen suggests an approximate 3.5-fold increased risk for Chinese women with endometriosis carrying the IL1RN*2-allele, we found this allele protective in Mexican women (except for endometriosis stage IV). The IL1RN*2-allele homozygote genotype has been associated with more prolonged and more severe proinflammatory immune response due to a decreased bioactivity/concentration of IL-1ra and with an increased production of IL-1β [56, 57]. The relationship between the IL-1RN genotype and its protein concentration has conflicting results because of the diversity of the environment, pathology and populations studied [57–60]. We do not know the biological meaning of a low frequency of IL1RN*2 allele and homozygote genotype in women with endometriosis. Nevertheless, the highest frequency of the IL1RN*2-allele homozygote genotype (25%) was observed again in endometriosis stage IV. This could lead to an insufficient production of IL-1Ra protein or to an overproduction of IL-1β in response to immune and/or inflammatory stimuli [60, 61], explaining why this allele could influence an individual’s susceptibility to endometriosis. Probably, this allele could genetically be associated with severity of disease (stage IV), rather than with predisposition [62]. Likewise, this could be related to an increased risk for intraperitoneal adhesion development [63], very common in severe stages of disease. A decreased production of IL-1Ra and an increase in IL-1β can contribute to a decrease in fibrinolysis during tissue repair [64].

To the best of our knowledge, the three polymorphisms evaluated in the present work are the first description of these genes in Mexican mestizo women with endometriosis. Although it appears that they are not associated with the risk of endometriosis, we identified that Mexican mestizo women with endometriosis stage IV have higher frequencies of TNF*2-, IL1B*2- and IL1RN*2-alleles than the CTR group, consistent with disease severity. Recent studies focused mainly on stage III/IV endometriosis, both recognized as severe stages of the disease, suggest a greater genetic burden when compared to stage I/II [65–67]. Genetic predisposition is an important factor in the etiology of endometriosis. Despite several genes and SNPs have been identified in the physiopathology and development [68, 69] of endometriosis, studies have demonstrated inconsistent and contradictory results due to its heterogeneous clinical manifestations and classification, research methodology, human genetic variability and different genetic ancestries and admixture. Thus, genetic research in endometriosis is complicated and has not been successful in providing replicable results.

A limitation of our study was the staging of endometriosis based on the rASRM score. Although it is the best well-known and the most used, it does not correlate well with clinical features and has drawbacks. Also, we did not include other associated genes and clustered polymorphism sites of lL-1, hence a haplotype analysis could not be done. A strength of our study is that all women (case and controls) were surgically evaluated for the presence/absence of endometriosis. More studies with larger sample sizes, well-matched controls, using other classifications of the disease (ENZIAN, Fertility score), considering more clustered polymorphisms, associated genes and other ethnic populations are necessary to definitively confirm the association of these polymorphisms reported by several studies. It would be necessary to evaluate in Mexican mestizo women other cytokine polymorphisms in the promoter region that have found associated to the disease [e.g. TNF (− 1031 T/C, − 863 C/A, − 857 C/T), IL-6 (− 634 T/C), or TGF-β (− 509 C/T)] [33]. Maybe these, among others, could help us explain the chronic local inflammation that has been related to the initiation, development and spread of endometriosis, as well as with the etiology of the symptoms, such as infertility and pain. Increasing the knowledge of genetic characteristics of inflammatory molecules in women with endometriosis from diverse ethnic groups will help us understand the onset and evolution of the phenomenon better.

## Conclusion

Although these three polymorphisms are not associated with the risk of endometriosis, Mexican mestizo women with severe stage of endometriosis (stage IV) have higher frequencies of TNF*2, IL1B*2- and IL1RN*2-alleles, which may explain a possible correlation with disease severity rather than predisposition or risk.

## Data Availability

The datasets generated and/or analysed during the current study are available in the ClinVar repository with the following accession numbers (web links) to datasets: SCV002073726 (https://www.ncbi.nlm.nih.gov/clinvar/?term=SCV002073726 [clv_acc]), SCV002073727 (https://www.ncbi.nlm.nih.gov/clinvar/?term=SCV002073727 [clv_acc]) and SCV002073728 (https://www.ncbi.nlm.nih.gov/clinvar/?term=SCV002073728 [clv_acc]).

## Abbreviations

CTR: Control group
CI: Confidence interval
ENDO: Endometriosis group
IL-1β: Interleukin-1beta
IL1B*1: IL-1β wild-type allele
IL1B*2: IL-1β mutated allele
IL-1Ra: Interleukin-1 receptor antagonist
IL1RN*1: IL-1Ra allele 1
IL1RN*2: IL-1Ra allele 2
IL1RN*3: IL-1Ra allele 3
IL1RN*4: IL-1Ra allele 4
IL1RN*5: IL-1Ra allele 5
INPer: Instituto Nacional de Perinatología
OR: Odds ratio
rASRM: Revised American Society of Reproductive Medicine
SNP: Single nucleotide polymorphisms
TNF-α: Tumor necrosis factor-alpha
TNF*1: TNF-α wild-type allele
TNF*2: TNF-α mutated allele
VNTR: Variable number tandem repeat
